# Supplementary material for: The phylogenetic landscape and nosocomial spread of the multidrug-resistant opportunist Stenotrophomonas maltophilia
Source: Nat Commun. 2020 Apr 27;11:2044. doi: 10.1038/s41467-020-15123-0 (PMC7184733; doi:10.1038/s41467-020-15123-0)
Supplement: Supplementary file 1 — Supplementary Information [file 41467_2020_15123_MOESM1_ESM.pdf]

Supplementary Information

The phylogenetic landscape and nosocomial spread of the multidrug-resistant opportunist *Stenotrophomonas maltophilia*

by Gröschel MI *et al.*

Correspondence to: sniemann@fz-borstel.de

This PDF file includes:

Supplementary Figures 1 to 8

Supplementary Tables 1 to 4

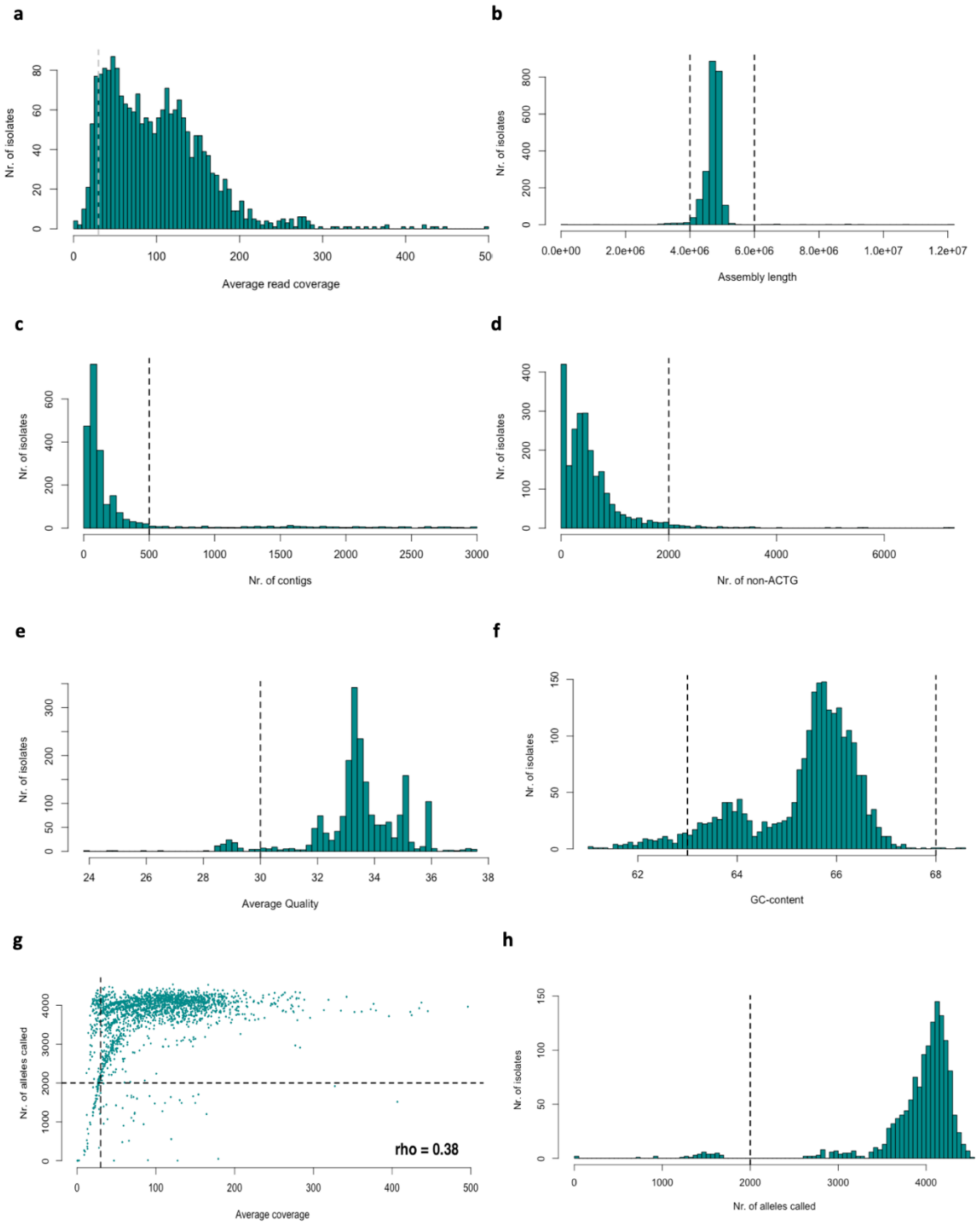

Supplementary Figure 1. Quality metrics upon assembly of a collection of 2,389 *S. maltophilia* complex genome datasets before filtering. Dashed lines indicate the quality thresholds applied in this study. Barplots. **a,** average read coverage. **b,** genome lengths

upon assembly. **c,** number of contigs. **d,** number of non-ACTG bases called. **e,** average quality. **f,** GC-content. **g,** Scatterplot of the number of allele calls received by the isolates versus coverage (Spearman's rank correlation coefficient shown on the right lower side of the figure). **h,** number of loci that were called and received an allele number of the wgMLST scheme. Source data are provided as Supplemental Data Files.

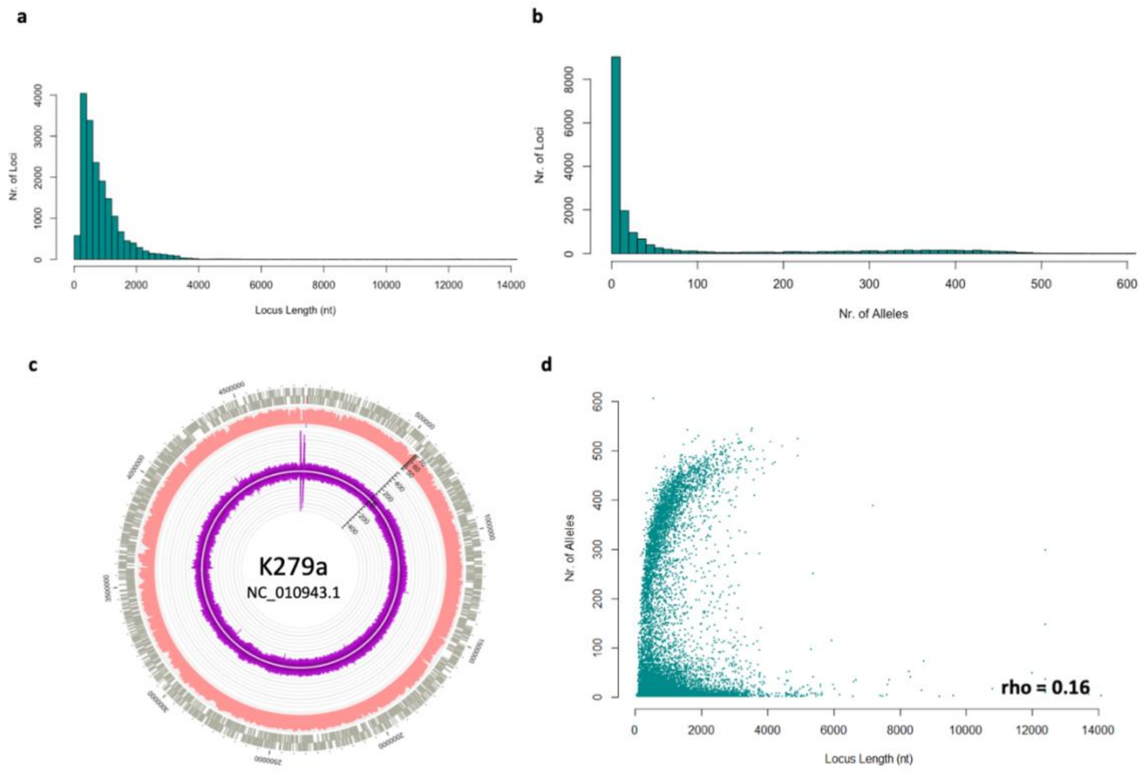

Supplementary Figure 2. Characteristics of the 17,603 loci of the wgMLST scheme constructed for the *S. maltophilia* complex. **a,** Distribution of the wgMLST loci lengths. **b,** Distribution of the number of different alleles per locus. **c,** Location of the wgMLST loci that map to the genome of strain *S. maltophilia* K279a. Coverage is depicted in purple, GC content in red. **d,** Impact of locus length on allele diversity (n = 1,305 genomes). Scatter plot of number of alleles versus locus length (Spearman's rank correlation coefficient shown on the right lower side of the figure). Source data are provided in Supplemental Data Files.

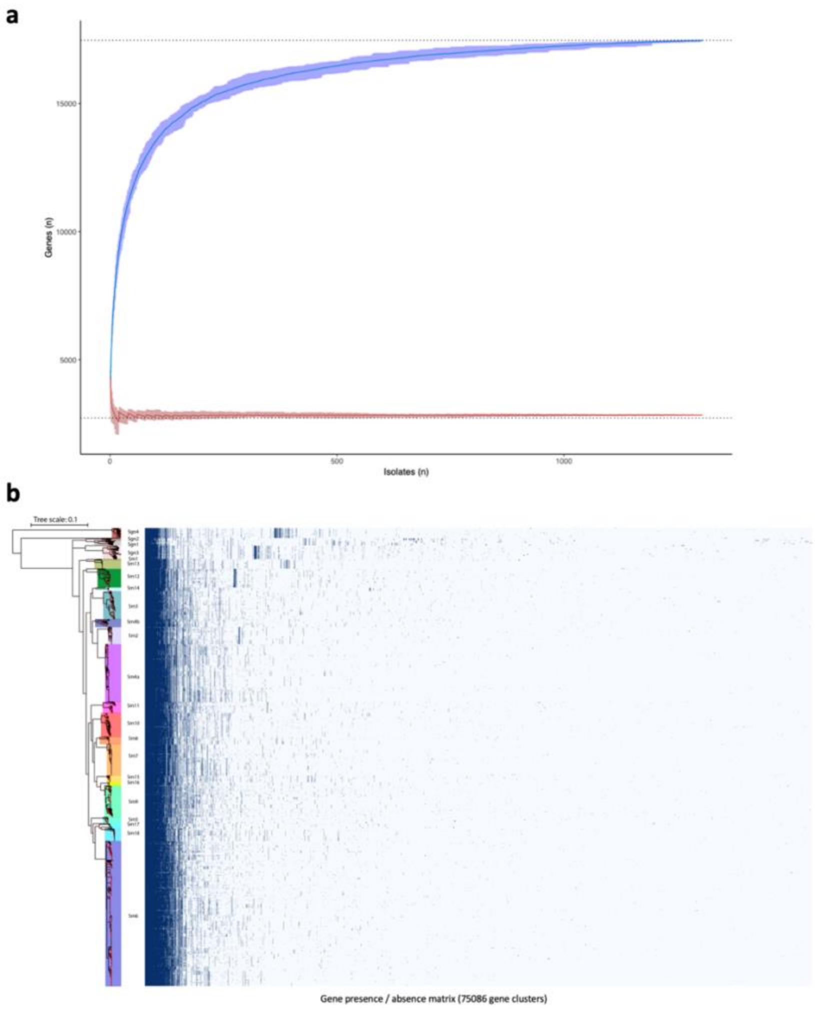

Supplementary Figure 3. Characteristics of the accessory genome. a, Rarefaction curve of the core- and pangenome of the 1,305 *S. maltophilia* complex strains in the study collection. Analysis based on the presence of genes in the assemblies and independent of the number of received allele calls for the respective loci of the wgMLST scheme. The x axis shows the number of strains taken into the analysis and the y axis displays the number of genes detected in the assembled genomes of these strains. Upon 100 repeats of random selection of genomes from the complete set, the minimum and maximum of each calculation are shown in shaded colours with the average as solid line. Blue line represents the pan genome, red line the 95% core genome. **b,** Gene presence/absence matrix displaying orthologous gene clusters arranged to the topology of the midpoint rooted phylogenetic tree on the left side. The matrix displays 75086 gene clusters. Source data are provided as Source Data Files.

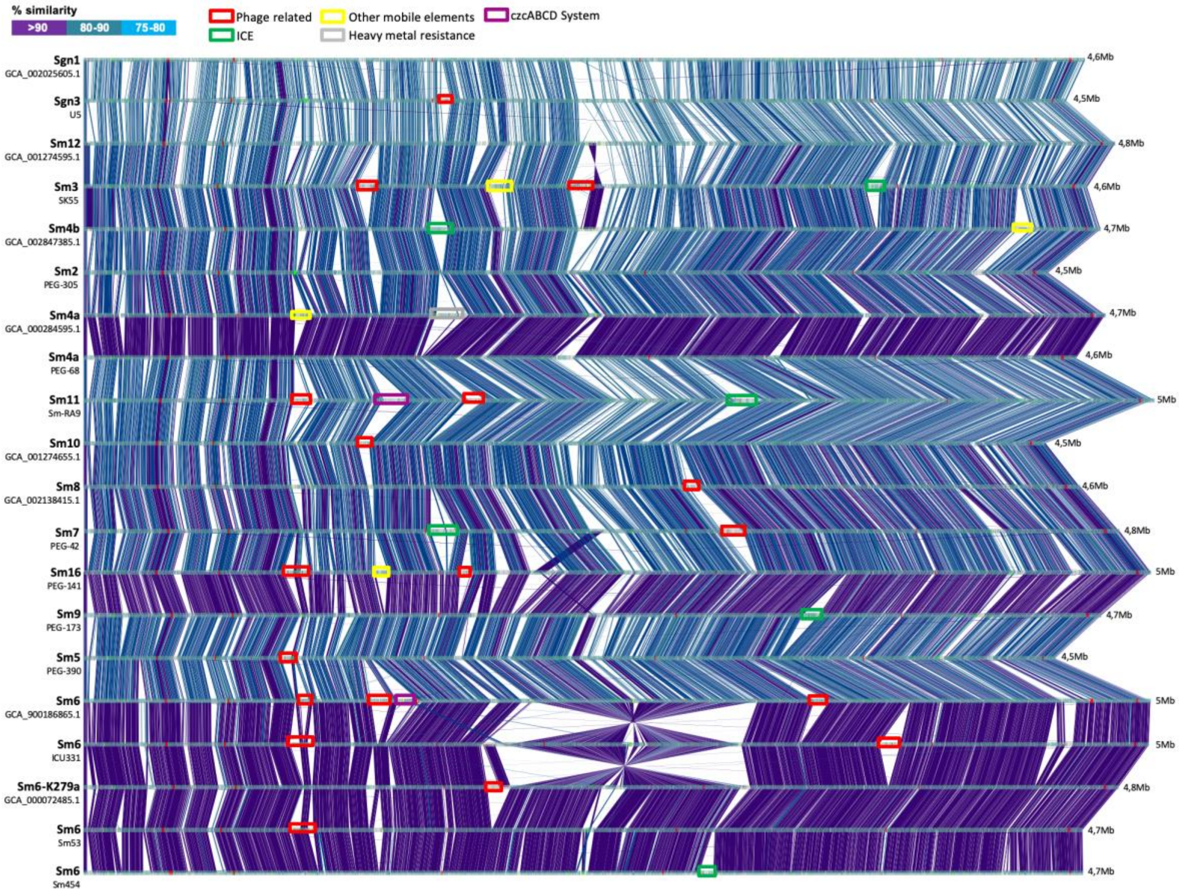

Supplementary Figure 4. **Structural variation indicated by a genome-wide alignment of 20 *S. maltophilia* complex genomes using blastn**. These genomes are representative of the 15 major phylogenetic lineages and include the strain K279a (accession NC_010943.1). The alignment shows major structural variation and different genome lengths of strains from different lineages and even for strains from the same lineage for lineages Sm4a and Sm6. One strain, ICU331, exhibited a large inversion of ~1Mb, as verified upon aligning the reads on the assembly and flanked by several IS elements. Colored squares show genomic islands found across the genomes. ICE = Integrative and conjugative elements.

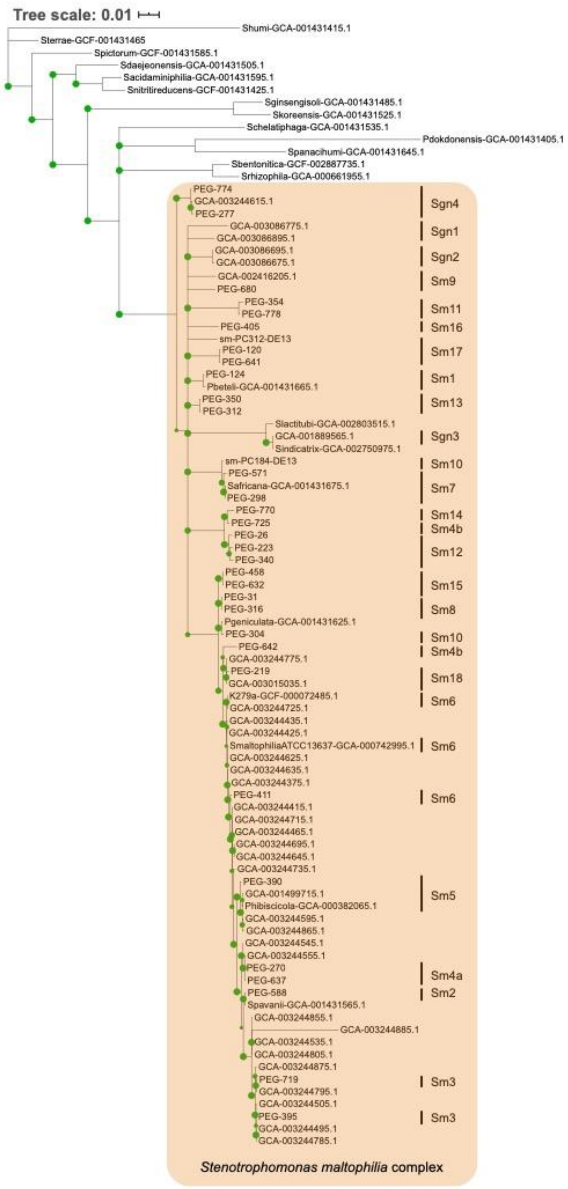

Supplementary Figure 5. **Comparative phylogenetic analysis of selected *S. maltophilia* complex isolates**. *S. maltophilia* isolates, representative of the lineages found in this study, were selected along with *Stenotrophomonas* species genomes and isolates from a recent taxonomic study by Patil P and colleagues (REF 15 main manuscript). Unrooted maximum likelihood phylogenetic tree calculated from the concatenated predicted amino acid sequences of 23 reference proteins. Orange shading highlights the *S. maltophilia* complex. Isolates from this study are marked with their respective lineage. Green dots represent bootstrap values of 1000 replicates. All nodes with bootstrap support below 70 are collapsed.

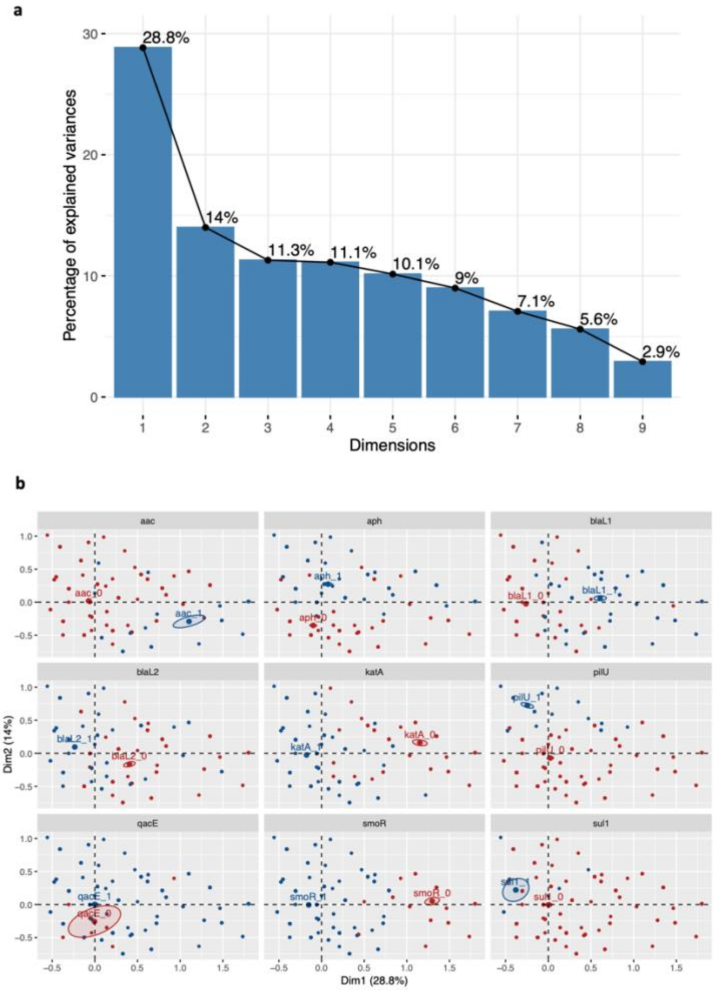

Supplementary Figure 6. Multiple correspondence analysis of 9 resistance or virulence genes. a, Barplot displaying the percentage of variance explained by the respective principal component dimensions of the multiple correspondence analysis (MCA). The first four dimensions provide the percentage of variance explained by the model while the rest are individual variations of active variables. **b,** MCA results shown on individual basis and grouped by each of the 9 resistance or virulence associated genes ((*aac, aph, blaL1, blaL2, katA, pilU, qacE, smoR, sul1*) acting as active variables. Presence/absence of a gene within an individual denoted with blue (= present) and red (= absent) points respectively. Source data are provided as Supplemental Data Files.

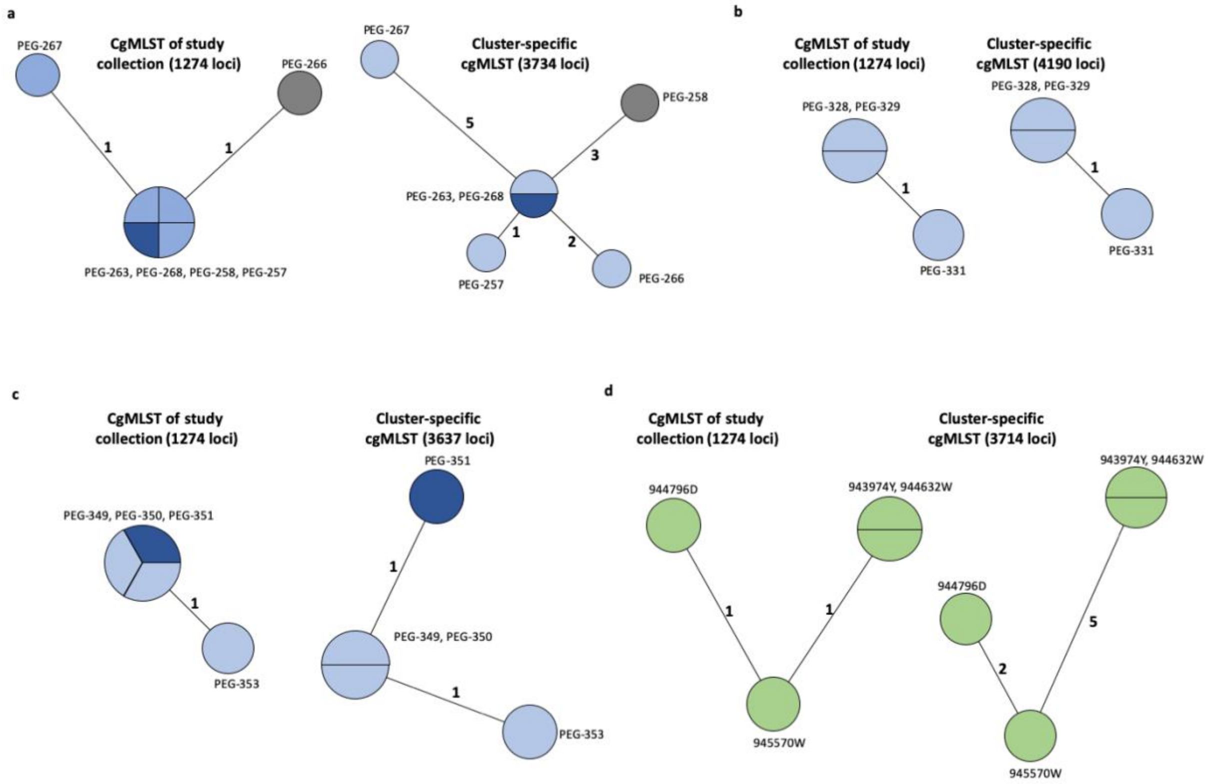

Supplementary Figure 7. Minimum Spanning Trees comparing the 1,275 study collection core genome MLST loci against the cluster specific core genome MLST loci as indicated above the respective trees. The use of cluster specific core MLST schemes as a sub-selection of the whole genome MLST increases resolution during outbreaks and demonstrates the flexibility of the wgMLST approach. **a,** Cluster 42 (hospital A). **b,** Cluster 45 (hospital B). **c,** Cluster 47 (hospital C). **d,** Cluster 52 (hospital D). The number of mismatched alleles are shown in small numbers on the connecting branches. Node colours indicate isolation source, light blue = respiratory sample, dark blue = sputum, grey = wound swap, green = endoscope. Source data are provided as Source Data Files.

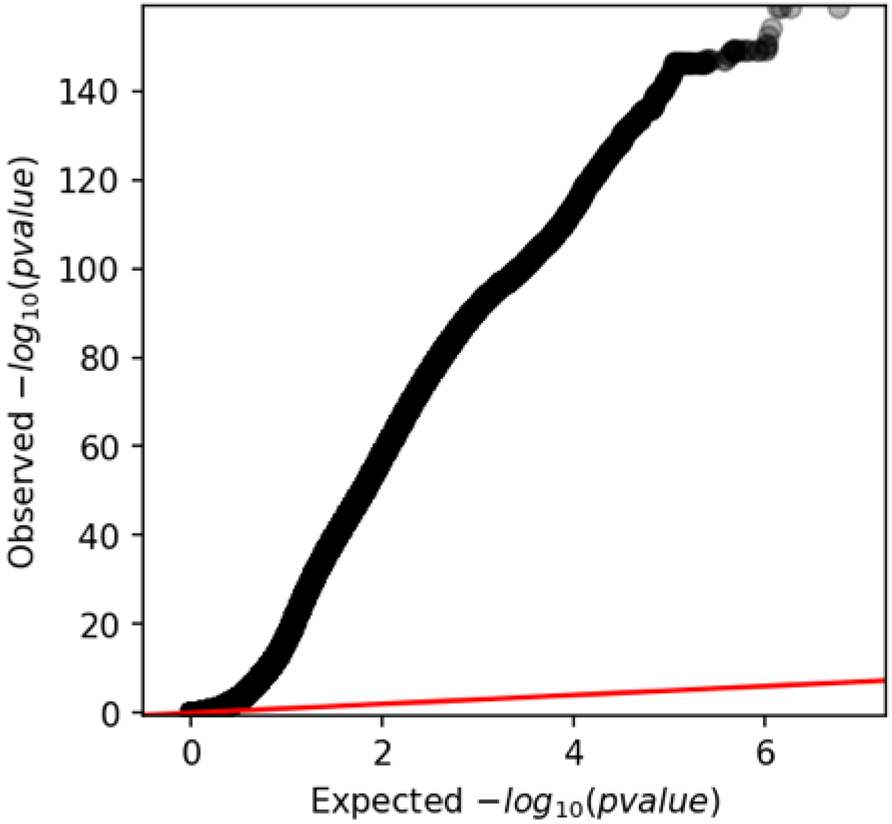

Supplementary Figure 8. Quantile-Quantile (Q-Q) plot of GWAS analysis of human-association versus environmental source using a linear mixed univariate model. The Q-Q plot shows the log transformed quantiles of the p-values to assess the probable significance of association between the genotype and the trait. The inflated nature of the slope strongly indicates substantial deviation between the observed and expected p-values due to a substantial genetic relatedness in the *S. maltophilia* dataset. A linear mixed model of fixed and random effects as implemented in pyseer was employed using a core phylogeny derived similarity matrix to control for population structure.

Supplementary Table 1. Details of the 7 classical Multilocus Sequence Typing loci (available at https://pubmlst.org/smaltophilia/ [accessed February 10th, 2019])

Gene name	Length	Min length (bp)	Max length (bp)	wgMLST loci (MLST sequence)	wgMLST loci (full sequence)
atpD	Fixed	531	531	STENO_17597	STENO_3197
gapA	Variable	558	561	STENO_17598	STENO_2953
guaA	Fixed	552	552	STENO_17599	STENO_1653
mutM	Fixed	465	465	STENO_17600	STENO_47
nuoD	Fixed	444	444	STENO_17601	STENO_2646
ppsA	Variable	492	495	STENO_17602	STENO_2281
recA	Fixed	546	546	STENO_17603	STENO_1423

Supplementary Table 2. Accession numbers, isolation source, and sequence metrics of 20 fully finished genomes from the major lineages, 12 of which have been sequenced within this study.

Isolate name	Accession	Lineage	Sequenced	Source	Mean read length (bp)	Mean coverage	No. of contigs	Length (Mb)	No. of genes	Plasmids
sm454	CP040431	Sm6	this study	human	12822	200	1	4,68	4210	0
ICU331	CP040440	Sm6	this study	human	9119	135	1	4,90	4563	0
SKK55	CP040433	Sm3	this study	human	11162	142	1	4,67	4206	0
PEG-141	CP040439	Sm16	this study	human	12112	116	1	5,00	4605	0
PEG-42	CP040435	Sm7	this study	human	12863	154	2	4,87	4427	0
PEG-173	CP040438	Sm9	this study	human	10384	144	1	4,76	4238	0
PEG-68	CP040434	Sm9	this study	human	8830	150	1	4,64	4143	0
PEG-305	CP040437	Sm2	this study	human	13952	165	1	4,50	3987	0
U5	CP040429	Sgn3	this study	environmental	13269	125	3	4,50	4064	0
PEG-390	CP040436	Sm5	this study	human	12253	183	1	4,55	4119	0
sm-RA9	CP040432	Sm11	this study	environmental	12190	132	1	5,00	4471	0
Sm53	CP040430	Sm6	this study	human	7678	119	1	4,68	4199	0
GCA_002025605.1	CP018756.1	Sgn1	NCBI	environmental			1	4,67		0
GCA_001274595.1	CP011010.1	Sm12	NCBI	human			1	4,8		0
GCA_002847385.1	CP025298.1	Sm4b	NCBI	unknown			1	4,74		0
GCA_000284595.1	HE798556.1	Sm4a	NCBI	unknown			1	4,77		0
GCA_001274655.1	CP011305.1	Sm10	NCBI	human			1	4,51		0
GCA_002138415.1	CP015612.1	Sm8	NCBI	unknown			1	4,67		0
GCA_900186865.1	LT906480.1	Sm6	NCBI	unknown			1	5		0
GCA_000072485.1	AM743169.1	Sm6	NCBI	human			1	4,85		0

Supplementary Table 3. Number of strains per lineage and their association with isolation source where known.

	Anthropogenic		Environmental		Human-invasive		Human-non-invasive		Human-respiratory		total per group
	n	p-value	n	p-value	n	p-value	n	p-value	n	p-value	
Sgn1	0	0.62	12	<.001	0	0.50	0	0.08	0	0.01	12
Sgn2	0	0.82	5	<.001	0	0.60	0	0.47	0	0.24	5
Sgn3	2	0.58	28	<.001	0	0.08	3	0.02	4	<.001	37
Sgn4	1	0.74	5	0.29	3	0.59	9	0.52	6	0.20	24
Sm1	0	0.91	0	0.83	1	0.49	1	0.59	0	0.52	2
Sm10	1	0.50	5	0.54	8	0.55	18	0.58	30	0.52	62
Sm11	7	<.001	11	<.001	3	0.62	5	0.34	3	<.001	29
Sm12	9	<.001	3	0.52	5	0.59	13	0.54	18	0.47	48
Sm13	0	0.54	0	0.34	2	0.59	4	0.46	17	0.03	23
Sm14	1	0.52	2	0.47	1	0.65	2	0.60	2	0.50	8
Sm15	2	0.47	0	0.47	1	0.54	6	0.58	9	0.54	18
Sm16	1	0.52	0	0.56	1	0.65	1	0.51	5	0.51	8
Sm17	0	0.60	0	0.51	3	0.47	6	0.47	4	0.50	13
Sm18	3	0.47	1	0.47	3	0.58	9	0.56	17	0.51	33
Sm2	0	0.47	1	0.33	4	0.59	9	0.47	26	0.04	40
Sm3	2	0.54	5	0.52	9	0.54	23	0.52	30	0.58	69
Sm4a	6	0.58	4	<.001	21	0.47	65	<.001	57	0.17	153
Sm4b	0	0.54	1	0.54	7	0.02	5	0.54	8	0.54	21
Sm5	1	0.60	5	0.06	3	0.52	4	0.54	4	0.28	17
Sm6	8	0.06	19	<.001	42	0.58	107	0.54	190	<.001	366
Sm7	2	0.52	3	0.17	7	0.52	29	0.47	40	0.49	81

Sm8	0	0.54	0	0.44	0	0.34	12	0.03	8	0.56	20
Sm9	6	0.47	6	0.50	8	0.54	21	0.47	45	0.33	86
ungrouped	0	0.84	1	0.54	1	0.54	1	0.68	1	0.54	4
Total per origin	52		117		133		353		524		1179

Note: Either the test of equal or given proportions or, for small sample sizes (n < 5), Fisher's exact test (both one-sided) was used in 124 comparisons while controlling the false discovery rate of multiple testing using the Benjamini-Hochberg procedure. * < .05; ** < .01; *** < .001

Supplementary Table 4. Clustering characteristics of the 1,305 *S. maltophilia* complex strains of the study collection divided by lineage based on 100 (d100 clusters) or 10 allelic mismatches (d10 clusters).

Lineage (n)	d100 clustering rate	d10 clustering rate	No. of d10 clusters	No. of d10 clustered isolates	No. of isolates in individual d10 cluster
Sgn1 (14)	0	0	-	-	-
Sgn2 (6)	0	0	-	-	-
Sgn3 (38)	0	0	-	-	-
Sgn4 (28)	0	0	-	-	-
Sm1 (2)	0	0	-	-	-
Sm2 (49)	0.59	0.31	3	15	3 - 8
Sm3 (81)	0.28	0.21	4	17	3 - 6
Sm4a (164)	0.83	0.18	8	30	3 - 4
Sm4b (22)	0.32	0.27	1	6	6
Sm5 (18)	0.5	0.38	2	7	3 - 4
Sm6 (413)	0.73	0.21	22	88	3 - 9
Sm7 (90)	0.92	0.27	4	24	3 - 12
Sm8 (21)	0.71	0.38	1	8	8
Sm9 (91)	0.42	0.21	4	19	3 - 10
Sm10 (67)	0.19	0.18	2	12	3 - 9
Sm11 (32)	0.22	0.22	2	7	3 - 4
Sm12 (53)	0.36	0.08	1	4	4
Sm13 (25)	0.88	0.48	2	12	6
Sm14 (9)	0.66	0.44	1	4	4
Sm15 (19)	0.74	0	-	-	-
Sm16 (9)	0.88	0.44	1	4	4
Sm17 (14)	0.64	0.29	1	4	4
Sm18 (36)	0.75	0.25	3	9	3
