# Supplementary material for: The phylogenetic landscape and nosocomial spread of the multidrug-resistant opportunist Stenotrophomonas maltophilia
Source: Nat Commun. 2020 Apr 27;11:2044. doi: 10.1038/s41467-020-15123-0 (PMC7184733; doi:10.1038/s41467-020-15123-0)
Supplement: Supplementary file 3 — Reporting Summary [file 41467_2020_15123_MOESM3_ESM.pdf]

nature research

Corresponding author(s): Stefan Niemann

Last updated by author(s): Feb 7, 2020

Reporting Summary

Nature Research wishes to improve the reproducibility of the work that we publish. This form provides structure for consistency and transparency in reporting. For further information on Nature Research policies, see Authors & Referees and the Editorial Policy Checklist .

Statistics

For all statistical analyses, confirm that the following items are present in the figure legend, table legend, main text, or Methods section.

n/a	Confirmed	
[ ]	[x]	The exact sample size (*n*) for each experimental group/condition, given as a discrete number and unit of measurement
[x]	[ ]	A statement on whether measurements were taken from distinct samples or whether the same sample was measured repeatedly
[ ]	[x]	The statistical test(s) used AND whether they are one- or two-sided<br>*Only common tests should be described solely by name; describe more complex techniques in the Methods section.*
[x]	[ ]	A description of all covariates tested
[ ]	[x]	A description of any assumptions or corrections, such as tests of normality and adjustment for multiple comparisons
[x]	[ ]	A full description of the statistical parameters including central tendency (e.g. means) or other basic estimates (e.g. regression coefficient) AND variation (e.g. standard deviation) or associated estimates of uncertainty (e.g. confidence intervals)
[ ]	[x]	For null hypothesis testing, the test statistic (e.g. *F*, *t*, *r*) with confidence intervals, effect sizes, degrees of freedom and *P* value noted<br>*Give P values as exact values whenever suitable.*
[x]	[ ]	For Bayesian analysis, information on the choice of priors and Markov chain Monte Carlo settings
[ ]	[x]	For hierarchical and complex designs, identification of the appropriate level for tests and full reporting of outcomes
[ ]	[x]	Estimates of effect sizes (e.g. Cohen's *d*, Pearson's *r*), indicating how they were calculated

Our web collection on statistics for biologists contains articles on many of the points above.

Software and code

Policy information about availability of computer code

Data collection	The Microsoft Office suite was used by hospitals to collect metadata and de-identified codes of the bacterial isolates.
Data analysis	Bionumerics v7.6.3<br>BWA v0.7.12<br>SPAdes v3.7.1<br>PlasmidFinder v2.1<br>NCBI blast v2.4.0+<br>InterProScan 5 online<br>RAxML-NG v0.9.0<br>ClonalFrameML v1.11<br>Maskrc-svg v.0.5<br>fastANI v.1.3<br>checkM-genome v1.1.1<br>Prokka v.1.14.5<br>Roary v.3.12.0<br>Abricate v.0.8.7<br>Pyseer v1.3.3<br>Unitig-counter v1.0.5<br>IQtree v1.6.12<br>blast2go available at https://www.blast2go.com/<br>itol v5 available at https://itol.embl.de/<br>R v.3.4.3<br>R package tidyverse v1.3.0

R package rhierBAPS v1.1.3
R package FactoMineR v2.0
R package factoextra v1.0.6
R package reshape2 v1.4.3
R package rcompanion v2.3.7
R package tmap v2.3-1
R package qqman v0.1.4

Code availability: Custom python scripts used in the phylogenetic analyses are available from https://github.com/conmeehan/pathophy. Custom R scripts used to calculate the statistical tests and perform the MCA analysis in Figs 3b-d and Supplementary Figure 5 are available from https://github.com/ngs-fzb/SMaltophilia_phylo and can be replicated using the source data files provided.

For manuscripts utilizing custom algorithms or software that are central to the research but not yet described in published literature, software must be made available to editors/reviewers. We strongly encourage code deposition in a community repository (e.g. GitHub). See the Nature Research guidelines for submitting code & software for further information.

Data

Policy information about availability of data

All manuscripts must include a data availability statement. This statement should provide the following information, where applicable:

- Accession codes, unique identifiers, or web links for publicly available datasets
- A list of figures that have associated raw data
- A description of any restrictions on data availability

Data availability: The sequencing data generated and analyzed during the current study are available in the European Nucleotide Archive repository under the accession numbers PRJEB32355, PRJEB32585, and PRJNA543082 (also see Supplementary Table 2). The Bacterial Antimicrobial Resistance Reference Gene Database is available under accession "PRJNA313047. All data generated or analysed during the current study are included in this published article and its supplementary information. The source data underlying Figs 1a-c, 3, 4a, 5a, Supplementary Figures 3b and 6 are provided as a Source Data File, along with supplemental Supplementary Data 3 and 5. Supplementary Data 3 contains the sample metadata used to create Figs 1d, 2, 5, and the tree annotation in Fig 4a. Supplementary Data 5 comprises the allelic typing information for all samples and was used to create Figs 4b, 5, and S7. The Supplementary Figures 1 and 2 are based on data provided in Supplementary Data 4 and Supplementary Data 2, respectively. The wgMLST scheme is available from https://figshare.com/articles/Smaltophilia_wgMLST_all-alleles_fasta_gz/10005047.

Field-specific reporting

Please select the one below that is the best fit for your research. If you are not sure, read the appropriate sections before making your selection.

☐ Life sciences ☐ Behavioural & social sciences ☒ Ecological, evolutionary & environmental sciences

For a reference copy of the document with all sections, see nature.com/documents/nr-reporting-summary-flat.pdf

Ecological, evolutionary & environmental sciences study design

All studies must disclose on these points even when the disclosure is negative.

Study description	This study represents a molecular epidemiological mapping of the opportunistic pathogen Stenotrophomonas maltophilia with an analysis of its population structure, resistance and virulence gene, clonal complexes and potential hospital outbreaks.
Research sample	All 1,305 Stenotrophomonas maltophilia complex strains included in this study were routinely collected in the participating hospitals across Germany, Switzerland, and Austria.
Sampling strategy	These strains were routinely collected by the participating clinical microbiology departments in the hospital. No prior sample size calculation was performed.
Data collection	Stenotrophomonas maltophilia isolates were identified using MALDI-TOF MS. Metadata was noted by participating hospitals on Microsoft Excel sheets that were anonymized and shared with the study team.<br><br>We further included all publicly available S. maltophilia sequence read sets up to April 2018.
Timing and spatial scale	Hospitals across Germany, Switzerland, and Austria participated during the years. We included strains collected up to 2017.
Data exclusions	Upon next generation sequencing, we excluded several hundreds strains from our study collection (including sequence data downloaded from the public domain). Detailed quality criteria are specified in the methods section of the manuscript.
Reproducibility	Each analysis was performed multiple times. Code along with source data and supplementary data files are provided to reproduce the findings of the study.
Randomization	Strains were considered environmental (n = 117) if found in natural environments e.g. in the rhizosphere, and anthropogenic if swabbed in human surroundings such as patient room sink or sewage (n = 52). Human-invasive (n = 133) was used for isolates from

blood, urine, drainage fluids, biopsies, or in cerebrospinal fluid, human-non-invasive (n = 353) refers to colonizing isolates from swabs of the skin, perineum, nose, oropharynx, wounds as well as intravascular catheters, and human-respiratory (n = 524) includes strains from the lower respiratory tract below the glottis and sputum collected from cystic fibrosis patients. For 126 strains, no information on their isolation source was available and, thus, these were not included in this analysis

Blinding

S. maltophilia strains were routinely collected from participating hospitals and diagnosed as such, so no blinding was involved in this study.

Did the study involve field work? ☐ Yes ☒ No

Reporting for specific materials, systems and methods

We require information from authors about some types of materials, experimental systems and methods used in many studies. Here, indicate whether each material, system or method listed is relevant to your study. If you are not sure if a list item applies to your research, read the appropriate section before selecting a response.

Materials & experimental systems

n/a	Involved in the study	
☒	☐	Antibodies
☒	☐	Eukaryotic cell lines
☒	☐	Palaeontology
☒	☐	Animals and other organisms
☒	☐	Human research participants
☒	☐	Clinical data

Methods

n/a	Involved in the study	
☒	☐	ChIP-seq
☒	☐	Flow cytometry
☒	☐	MRI-based neuroimaging
