# Supplementary material for: The phylogenetic landscape and nosocomial spread of the multidrug-resistant opportunist Stenotrophomonas maltophilia
Source: Nat Commun. 2020 Apr 27;11:2044. doi: 10.1038/s41467-020-15123-0 (PMC7184733; doi:10.1038/s41467-020-15123-0)
Supplement: Supplementary file 4 — Description of Additional Supplementary Files [file 41467_2020_15123_MOESM4_ESM.pdf]

Description of Additional Supplementary Files

File Name: Supplementary Data 1
Description: List of NCBI GeneBank accession numbers of the 171 assembled genomes used to create the whole-genome Multilocus sequence typing scheme.

File Name: Supplementary Data 2
Description: Characteristics of the 17,603 loci of the whole-genome Multilocus Sequence Typing scheme.

File Name: Supplementary Data 3
Description: Accession numbers and associated metadata for all 1,305 S. maltophilia isolates included in the phylogenetic analysis.

File Name: Supplementary Data 4
Description: Sequence metrics, number of consensus whole genome Multilocus sequence typing allele calls of all 1305 S. maltophilia isolates and other Stenotrophomonas species.

File Name: Supplementary Data 5
Description: Valid allele call numbers assigned by whole genome multilocus sequence typing scheme as implemented in bionumerics for all 1,305 S. maltophilia isolates.
